# Ethno-National Attitudes as Intercultural Competence Predictors in University Students: Gender Differences

**DOI:** 10.3390/bs10020056

**Published:** 2020-02-06

**Authors:** Irina A. Novikova, Marina V. Gridunova, Alexey L. Novikov, Dmitriy A. Shlyakhta

**Affiliations:** Social and Differential Psychology Department, Peoples’ Friendship University of Russia (RUDN University), 6 Miklukho-Maklaya Str., Moscow 117198, Russia

**Keywords:** intercultural competence, intercultural sensitivity, developmental model of intercultural sensitivity, ethno-national attitudes, university students, gender differences

## Abstract

The search for predictors of intercultural competence (ICC) development is one of the important challenges of modern psychology in connection with globalization in all the spheres of modern life, including university education. The purpose of the present research is to show that the ethno-national attitudes (ENA), which Khukhlaev et al. consider as an individual’s predisposition to assess the nationality/ethnicity can determine the severity of ICC features in male and female university students. The sample includes 219 (75% female) first–third year Russian university students. ICC was measured with the author’s modification of the intercultural sensitivity scale (ISS) by Khuhlaev and Chibisova. ISS is based on the Developmental Model of Intercultural Sensitivity, (DMIS) by Bennett and includes 4 subscales: Minimization, Absolutization, Ambivalence, Acceptance. ENA (Nationalistic, Patriotic, Neutral, Negativistic) were determined with the *“Scale of ethno-national attitudes”* by Khukhlaev, Kuznetsov, and Tkachenko. Descriptive statistics methods, Wilcoxon *W*-test, and multiple regression analysis were used for statistical analysis in the R software environment, version 3.5.2. The findings of our research showed that Nationalistic and Patriotic ENA are the strongest predictors of studied ICC scales and have an opposite negative impact on *Acceptance* and *Absolutization* in both male and female students. This fact should be taken into account in the context of ICC developments.

## 1. Introduction

The development of intercultural competence (ICC) is one of the most important theoretical, applied, and practical psychological problems in the changing and globalizing world [1,2,3,4]. This problem is of particular relevance due to the growth of academic mobility in modern education and the increase in the number of international university students. For example, today 250,000 international students are studying in Russian universities and this number should increase threefold by 2025 [5].

Western and Russian scientists have developed more than 60 different models of the ICC [1,2,3,4,6,7,8] and similar concepts such as *intercultural communicative competence* [9,10], *intercultural sensitivity* [1,2,11,12], *cultural intelligence* [13,14], *multicultural effectiveness* [15], and some others [16]. The ICC concept, in our opinion, is not only the most frequently studied, but also the broadest of the listed above. Therefore, in present and other of our publications on this topic, we use the term *intercultural competence* (ICC) in the broadest sense, as a generalizing definition of the phenomenon characterizing the personality activity in a multicultural environment towards the different aspects of intercultural diversity and dialogue [17,18,19,20].

The Development Model of Intercultural Sensitivity (DMIS) by Bennett [2,21] is one of the most applied ICC models in modern psychology. DMIS proposes to consider the ICC as a process of changing of “the orientations toward cultural difference”: from ethnocentrism to ethnorelativism [2]. The first three stages of DMIS are ethnocentric and are characterized by the total *denial* of cultural differences, creating the *defense* from the differences and *minimizing* their influence. The following three ethnorelativistic stages imply *acceptance* of cultural differences, *adaptation* to their influence and *integration* of another worldview with one’s own [2]. An important advantage of DMIS is that Bennet and colleagues developed the Intercultural Development Inventory (IDI) for ICC measurement on its basis [2,22].

Russian psychologists Khukhlaev and Chibisova developed a Russian-language technique called the *intercultural sensitivity scale* (ISS) for ICC diagnostics based on the DMIS, which includes 4 subscales: *Minimization*, *Absolutization*, *Ambivalence*, and *Acceptance*. *Minimization* and *Absolutization* correspond to ethnocentric orientations according to the DMIS. *Ambivalence* is a transitional orientation from ethnocentrism to ethnorelativism. *Acceptance* corresponds to ethnorelative orientations according to the DMIS [7,23].

Numerous empirical studies revealed that the development of ICC is associated with various *socio-cultural*, *social-psychological, personality*, and *individual* determinants [12,15,16,23]. The *socio-cultural factors* of ICC (cultural diversity, multicultural context, length of stay in a multicultural environment, etc.) are fairly well studied on the basis of different models [15,23,24,25,26,27,28,29,30].

Among the *socio-psychological factors* of the ICC, various social attitudes are primarily studied [31,32,33]. We assume that one of the possible socio-psychological predictors of the ICC development is the attitudes towards the individual’s ethnicity. Despite the fact that the relationship and mutual influence of ethnic attitudes and ICC seems logical, the relevant research is almost not described in the scientific literature. One of the few examples is a study of the relations between ICC and ethnic attitudes towards Jewish couples in a small sample of Israeli midwives: it was found that the most positive attitudes and the lowest prejudice towards patients are observed in midwives if their religious identity coincided [33]. In Western psychology, starting with Allport [34], a lot of research has been done on the psychological predictors of prejudice [35,36] as well as relationships between ethnic attitudes and ethnic identity [37,38], which, however, are only indirectly related to the ICC predictors problem.

In Russian psychology, ethnic attitudes are actively studied in connection with the problem of nationalism and extremism. Khuhlaev proposes to consider “ethno-national attitudes” (ENA) as attitudes towards the phenomenon of “nationality” [39], which is synonymous to ethnicity in the current Russian public discourse [40]. Khuhlaev et al. distinguished four groups of ethno-national attitudes: Nationalistic (hostile attitudes towards people of other nationalities), Patriotic (feelings of pride for one’s own national identity and connection with people of “same nationality”), Neutral (indifferent attitude towards one’s own national identity), and Negativistic (negative attitude towards the phenomenon of nationality and national identity) [41,42]. Authors showed that nationalistic attitudes are positively connected with various forms of intolerance. Patriotic attitudes are linked with negative attitudes only towards other ethnic groups, as well as with positive attitudes towards own group. Neutral and negativistic ethno-national attitudes are positively connected with various types of tolerance (ethnic, social, personal trait) and attitudes towards other ethnic groups [42]. But ethno-national attitudes were not previously investigated as ICC predictors in Russian psychology. 

In our opinion, *personality* and *individual* determinants of ICC are less studied than socio-cultural and socio-psychological factors, especially in Russian psychology. Recently, in Western psychology, the influence of different personality traits on the ICC and related concepts has been actively studied [13,15,43,44,45,46]. In our previous research [17,18,19,20], we mainly investigated the personality factors of the ICC, considered on the Five-Factor Model (FFM) by McCrae and Costa [47,48]. We found that the majority of FFM personality traits correlate with the ICC indicators in Russian university and school students [17,18,19]. Namely, extraversion, openness, and conscientiousness are positively associated with *Acceptance*; extraversion and agreeableness with *Ambivalence*; and agreeableness with *Absolutization* in university students [17,19]. FFM personality traits, and above all conscientiousness and extraversion, are related to the ICC in secondary school students, but these relations vary in groups with different levels of intellectual development [18]. Further use of regression analysis allowed us to find that one or more of the FFM personality traits can explain only between 3.6% and 9.5% of the ICC variance considered on the basis of the DMIS in Russian university and school students. In particular, the FFM personality traits relatively more strongly affect the ICC features in school students (from 5.9% to 9.5% explained variance) than in university students (from 3.6% to 3.8% explained variance). On average, it was revealed that the relatively universal predictor of ICC features in both groups is ***consciousness***: it has a positive impact on *Minimization* in school students, a positive impact on *Acceptance* in university students, and a weak but negative impact on *Absolutization* (ethnocentric subscale) in both groups of students [20].

Possible *individual* predictors of the ICC include age and gender, which both are poorly researched. Most studies of the ICC problem have been performed on student and adult samples, while the characteristics of ICC in adolescents have not been sufficiently studied [20]. Insufficient attention to the study of gender differences in ICC may be due to the fact that, when Hammer, Bennett, and Wiseman developed and tested the first version of the IDI, they concluded that “it would appear that the IDI is not systematically influenced by gender differences” [2]. However, numerous studies of the gender specificity of human emotions indicate greater emotionality of women compared with men, which allows us to assume their greater sensitivity to intercultural differences. This assumption is supported by the conclusions by Kehl and Morris based on the literary review: female students are more susceptible to the impact of studying abroad, as their thinking becomes more global [45]. The opposite conclusions were reached by Wang and Ching, who showed that some indicators of intercultural effectiveness (“relaxation in interaction” and “management of interaction”) are higher among young men [49]. Using the Russian adaptation of the DMIS, Logashchenko revealed that male and female samples differ on the *Absolutization* scale: women are more confident than men in their ability to control the influence of cultural differences on the communication process [23]. In our previous study [20,50], we confirmed this conclusion regarding differences on the *Absolutization* scale, and also found gender differences on the Acceptance scale (the ability to notice and take into account intercultural differences is more characteristic of female university and school students) [20,50].

In summary, we emphasize once again that ICC development is determined by a combination of factors: socio-cultural, socio-psychological, personality, and individual, which must be studied together. Due to the fact that there are so few studies of the ICC based on the DMIS and its Russian modifications in Russian psychology, the research of any ICC predictors of different levels, for example socio-psychological (ENA) and individual (gender differences), is relevant.

Thus, the purpose of the present study is to consider the ethno-national attitudes as ICC predictors based on the DMIS and compare their impacts in male and female university students. 

Based on the ethno-national attitudes definition and the results of its research in the context of intercultural relations [39,41,42,51,52], we can assume that *Patriotic* ethno-national attitudes will have a positive impact on the ICC development (i.e., ethno-relativistic orientation prevalence). Although Khukhlaev et al. [39,41,42,52] showed a positive connection between Patriotic attitudes and above all with attitudes towards one’s own group, Manoilova [52] found connection between a positive attitude to one’s ethnic group and a positive attitude to other ethnic groups [51]. Based on these findings, we hypothesized that respect for native culture can be manifested in a broader context: as respect for the history, customs, and traditions of the mankind. *Nationalist* ethno-national attitudes, on the contrary, can become a barrier to the ICC development (i.e., ethnocentric orientation prevalence), because they are based on hostility to other cultures. The impact of *Neutral* and *Negativistic* ethno-national attitudes may be ambiguous, since, on the one hand, they reduce the probability of a conflict comparison of one’s own and other ethnic groups and therefore can positively influence the ICC development, but on the other hand, an indifferent and negative attitude to the nationality (ethnicity) can interfere with intercultural differences perception, which, in turn, reduces the possibility of ICC development. Based on our previous research of ICC [50], we also assume that there are differences in the degree of impact of ethno-national attitudes on ICC features in male and female students: in the male group this influence should be more pronounced.

## 2. Methods 

### 2.1. Participants

A total of 219 (75% female) university students, aged 17 to 24 (the mean is 19.06 ± 1.57 years) took part in the research. All of them were first-, second- and third-year students of different departments of two large Moscow universities (RUDN University and National University of Science and Technology MISiS). The students represent different regions of Russia, as well as the republics of the former USSR. All of them are ethnically Russian, speak Russian as a native language and study in Russian. They participated in the study during classes in psychological and pedagogical disciplines, as one of the elective tasks, for which they received additional points. They were advised that participation would be free and voluntary. 

All subjects gave their informed consent for inclusion before they participated in the study. The study was conducted in accordance with the APA Ethical Standards and the Code of Ethics of the RPS (Russian Psychological Society), and the protocol was approved by the Ethics Committee of RUDN University (# 050422-0-015).

### 2.2. Techniques

The *Intercultural Sensitivity Scale* (ISS) by Khukhlaev and Chibisova [7] in Logashchenko’ modified version [23] contains 51 items, which are grouped into four subscales (*Minimization, Absolutization, Ambivalence, Acceptance*), described in detail above. We used the author’s modifications of this technique with the following changes [17,18,19,20]:

(1) We asked respondents to express their degree of consent with items using a direct response Likert scale: from 0 (“totally disagree”) to 10 (“absolutely agree”), instead of a reverse scale that was used in the previous modification by Logashchenko;

(2) We reduced the number of items on each subscale to 8 based on a psychometric study using factor analysis, and the coefficients Cronbach’ *α* and McDonald’s *ω_h_*. All subscales of the ISS modified by us have acceptable Cronbach *α* coefficients (0.60–0.78) and McDonald *ω_h_* coefficients (0.47–0.76), which indicates their acceptable internal consistency [53,54]. In this ISS version the total raw scores for each subscale can range from 0 to 80 points.

The *ethno-national attitudes scale* (ENAS) by Khukhlaev, Kuznetsov, and Tkachenko [42] consists of 17 direct and inverted items to which the subject expresses the degree of consent on a 5-point Likert scale (from “strongly disagree” to “strongly agree”). The indicators for 4 subscales corresponding to the ENA described above (Nationalistic, Patriotic, Neutral, and Negativistic) are calculated using special formulas. The total raw scores for each subscale can range from 1 to 5 points.

Subscales have good internal consistency, as well as configurational and measurement invariance [42]. Example items for the *Nationalistic Attitudes* subscale are: (1) There are such nationalities, among which almost all people are bad; (2) I don’t like it if a person near me starts to speak a native (not our) language. Sample items from the *Patriotic Attitudes* subscale are: (1) When I think about my nationality, I feel pride and love; (2) I feel a kinship with people of my nationality. Some items from the *Neutral Attitudes* subscale include: (1) For me, my nationality plays no role in everyday life; (2) Nationality exists, but it is not very important. Finally, example items from the *Negativistic Attitudes* subscale are: (1) Separation of nationalities harms society; (2) It’s not very right to separate people according to nationality.

### 2.3. Statistical Analysis

The descriptive statistics methods, coefficients Cronbach’ *α* and McDonald’s *ω*, Wilcoxon rank sum test with continuity correction, Fisher *F*-test, and the multiple regression analysis were used for statistical analysis. The regression analysis by the method of “backward” stepwise search was used. Independent variables were the ethno-national attitudes (4 ENAS scales) and dependent variables were the ICC features (4 ISS subscales). In a first step, the full regression models with all possible predictors for each ISS subscale were constructed separately for male and female students. The next step involved analyzing all the input models by the method of search of all possible predictor combinations with evaluating the informational contribution of each set using the Akaike information criterion (AIC). Models having the highest information load at the smallest quantity of predictors (“a best predictor model”) were selected for further analysis. Statistical processing was carried out in the R software environment for statistical computing and graphics, version 3.5.2 [55].

## 3. Results 

Table 1 presents the results of descriptive statistics (means and standard deviations) and analysis of differences between all studied variables by *W*-test in male and female students. Significant differences exist only between Neutral attitudes, which are higher in male students (*p* = 0.049). 

The results of the multiple regression analysis (best predictor models) are presented in Table 2, Table 3, Table 4 and Table 5. The multiple correlation coefficients between the dependent variable and predictors for most of the models are statistically valid according to the Fisher *F*-test, which confirms that there is a significant impact of the ethno-national attitudes (ENA) on the ICC indicators. At the same time, there is a large range of the determination coefficients (*R*^2^), which reflects the different degree of the ENA influence on different ISS subscales.

Table 2 shows that the best predictor model for *Acceptance* predicts 10.4% of the variance in the general sample, 18.1% in male students and only 8.3% in female students. The significant predictors of *Acceptance* (ethno-relativistic subscale) in all studied samples are Nationalistic and Patriotic ENA, but its influence is opposite: Nationalistic ENA has a negative impact, while Patriotic ENA has a positive one. The revealed impacts completely correspond to our hypothesis and to the definition of Nationalistic and Patriotic ENA. It can be noted that both predictors have a stronger impact in male than in female students and this fact is also consistent with our assumptions. 

Table 3 shows that regression models for *Ambivalence* (transition stage from ethnocentrism to ethno-relativism) predict only by 3%–4% of the variance in the all samples. Differences between samples are that Patriotic ENA as a positive predictor is not significant in male students, while a significant model in female students includes Neutral ENA with negative impact. 

Table 4 shows that for *Absolutization* (ethnocentric subscale) the regression models with the highest determination coefficients were obtained (*R*^2^ = 0.336–0.356) in all studied samples. As expected, Nationalistic ENA have a strong positive impact on *Absolutization* that manifests itself in an exaggeration of the significance of cultural differences, in the attribution to the cultural characteristics of a stronger influence than they actually have, and in a purposeful search for cultural or ethnic attributes that favorably distinguish their own ethno-cultural group from others. Neutral (indifferent) ENA, on the contrary, have a negative impact on *Absolutization* and prevent the overestimation of cultural characteristics and differences. The only difference in the regression models between the studied samples is that Neutral ENA are not significant predictors in male students, despite the fact that they are higher in this group.

Table 5 shows that a significant regression model for *Minimization* (ethnocentric subscale) was obtained only in the general sample of university students and in female students. These models include Nationalistic and Patriotic ENA with weak negative impacts (*R*^2^ = 0.050–0.062).

## 4. Discussion

The purpose of the present study is to consider ethno-national attitudes as ICC predictors based on the DMIS, and compare their impacts in male and female university students. We surmise that ENA, especially Patriotic and Nationalistic, can predict the features of ICC in opposite ways and that their influence varies in male and female samples. Summarizing the results of the study, we can conclude that our hypothesis was mainly confirmed regarding the ENA impact on ICC, but less confirmed regarding gender differences.

Firstly, it was found that the ENA can explain from 3.1% to 35.6% of the ICC variance on the basis of the DMIS. Such determination coefficients are considered to be a not very high, yet the result can be deemed satisfactory, given the huge quantity of external and internal factors influencing the ICC indicators. Secondly, it was shown that most significant predictors for the ICC features are Nationalistic and Patriotic ENA with opposite impacts. As was expected, Nationalistic ENA has a positive impact on *Absolutization* (ethnocentric orientation toward cultural differences) and a negative impact on Acceptance (ethno-relativistic orientation toward cultural differences), and Patriotic ENA has a reverse effect in these cases. These ENA have the same impact on ICC in the general sample and in male and female students groups. Thirdly, minor differences in ENA impact on ICC features were identified between male and female university students, but they are not as significant as we expected. Regarding the lack of significant gender differences between the ICC scales, our data are consistent with the findings of Hammer et al. [2]. 

Thus, we can conclude that ENA are important predictors of ICC development stages from absolutization to acceptance of cultural differences. According to our data, ENA have a stronger impact on ICC than FFM personality traits [20]. Therefore, in order to increase the effectiveness of the ICC development programs, special attention should be paid not only to the students’ personality traits, but also to their socio-psychological characteristics and, above all, ethno-national attitudes, ethnic identity, stereotypes, prejudices, and the cultural position of students in multicultural environment.

There are several limitations to our study that should be taken into account when conducting future research in this area. Firstly, one limitation is the sample size and its female-to-male ratio. Secondly, only ethnically Russian students were involved in our study. We understand that both ethnic origin and ethnic identity are important factors of the ICC development, which must be taken into consideration in further studies. Another possible limitation of this study is the measure used to collect the data. The ICC can be measured with various tools, the most popular being self-questionnaires. However, there is a need to use more objective methods, including expert assessments and quasi-experiments. The next limitation is a certain lack of prior research studies on this topic in Russian psychology, so it is difficult to compare our results with other researchers’ and provide a more comprehensive outlook on the problem.

Summing up all the findings and limitations of our research, we can determine its future prospects: (1) the complex study of different ICC predictors (social and cultural context and experience; social attitudes, personality traits, intelligence level, creativity, national and/or cultural origin, ethnic identity, gender, age etc.); (2) a sample expansion and its balancing by the female-to-male ratio; (3) using of additional measurement methods for both ENA and ICC, as well as other methods of statistical analysis; (4) the development of the programs to improve the ICC in university students in a multicultural educational environment, taking into account the ICC predictors of different levels (socio-cultural, social-psychological, personality, and individual determinants).

## Figures and Tables

**Table 1:** Means (M), standard deviations (SD) and Wilcoxon rank sum test with continuity correction (*W*-test) between study variables in male and female Students.

Variable	General Sample (*N* = 219)		Male Students (*N* = 55)		Female Students (*N* = 164)		Wilcoxon’ *W*-Test	*p*-Level
	M	SD	M	SD	M	SD		
*Orientations toward cultural difference (ISS subscales)*								
Acceptance	56.50	12.16	54.24	13.20	57.26	11.73	5022.0	0.208
Ambivalence	33.85	14.30	36.55	14.73	32.95	14.08	3935.5	0.157
Absolutization	52.70	11.13	53.16	11.31	52.55	11.10	4370.5	0.732
Minimization	51.12	10.85	49.84	11.59	51.55	10.59	4776.0	0.513
*Ethno-national attitudes (ENAS subscales)*								
Nationalistic	2.28	0.82	2.25	0.77	2.30	0.84	4652.0	0.727
Patriotic	3.27	0.94	3.15	0.85	3.32	0.90	5091.0	0.152
Neutral	3.36	1.05	3.60	0.93	3.27	1.07	3714.0	0.049 *
Negativistic	2.98	0.40	2.97	0.46	2.99	0.38	4742.5	0.557

* *p* < 0.05. ISS: intercultural sensitivity scale.

**Table 2:** Best predictor regression models for ***Acceptance*** subscale of ISS.

*Sample/Variable*	*Summary of Model*			*Coefficients*			
	*R* ^2^	F	*p*-Value	Estimate	Std.Error	*t*-Value	*p*-Value
*General sample (N = 219)*	0.104	12.56	0.000				
(Intercept)				55.704	3.174	17.552	0.000
Nationalistic attitudes				−4.314	1.004	−4.298	0.000
Patriotic attitudes				3.254	0.869	3.743	0.000
*Male students (N = 55)*	0.181	5.758	0.006				
(Intercept)				56.628	7.131	7.941	0.000
Nationalistic attitudes				−7.119	2.259	−3.152	0.003
Patriotic attitudes				4.329	2.043	2.119	0.039
*Female students (N = 164)*	0.083	7.328	0.001				
(Intercept)				55.934	3.530	15.846	0.000
Nationalistic attitudes				−3.532	1.110	−3.182	0.002
Patriotic attitudes				2.846	0.954	2.983	0.003

**Table 3:** Best predictor regression models for ***Ambivalence*** subscale of ISS.

*Sample/Variable*	*Summary of Model*			*Coefficients*			
	*R* ^2^	F	*p*-Value	Estimate	Std.Error	*t*-Value	*p*-Value
*General sample (N = 219)*	0.031	6.965	0.009				
(Intercept)				45.895	2.684	17.098	0.000
Patriotic attitudes				2.080	0.788	2.639	0.009
*Male (N = 55)*	0.039	2.163	0.147				
(Intercept)				44.846	5.854	7.660	0.000
Patriotic attitudes				2.644	1.798	1.471	0.147
*Female (N = 164)*	0.032	5.394	0.022				
(Intercept)				58.630	2.754	21.287	0.000
Neutral attitudes				−1.858	0.800	−2.323	0.021

**Table 4:** Best predictor regression models for ***Absolutization*** subscale of ISS.

*Sample/Variable*	*Summary of Model*			*Coefficients*			
	*R* ^2^	F	*p*-Value	Estimate	Std.Error	*t*-Value	*p*-Value
*General sample (N = 219)*	0.336	54.6	0.000				
(Intercept)				22.035	4.029	5.469	0.000
Nationalistic attitudes				8.804	1.008	8.733	0.000
Neutral attitudes				−2.471	0.788	−3.138	0.002
*Male students (N = 55)*	0.338	13.27	0.000				
(Intercept)				26.148	9.371	2.790	0.007
Nationalistic attitudes				9.639	2.234	4.314	0.000
Neutral attitudes				−3.128	1.839	−1.701	0.095
*Female students (N = 164)*	0.356	44.48	0.000				
(Intercept)				22.142	4.400	5.033	0.000
Nationalistic attitudes				8.526	1.110	7.680	0.000
Neutral attitudes				−2.680	0.865	−3.097	0.002

**Table 5:** Best predictor regression models for ***Minimization*** subscale of ISS.

*Sample/Variable*	*Summary of Model*			*Coefficients*			
	*R* ^2^	F	*p*-Value	Estimate	Std.Error	*t*-Value	*p*-Value
*General sample (N = 219)*	0.050	5.699	0.004				
(Intercept)				60.220	2.916	20.655	0.000
Nationalistic attitudes				−2.221	0.922	−2.409	0.017
Patriotic attitudes				−1.229	0.799	−1.539	0.125
*Female students (N = 164)*	0.062	5.312	0.006				
(Intercept)				61.512	3.222	19.090	0.000
Nationalistic attitudes				−2.109	1.013	−2.081	0.039
Patriotic attitudes				−1.543	0.871	−1.771	0.078

## Data Availability

The datasets used and analyzed during the current study are available from the corresponding author upon reasonable request.
